# Can Molecular Response Redefine Organ Preservation in Rectal Cancer? From Clinical Complete Response to Precision Watch-and-Wait

**DOI:** 10.3390/medicina62101865

**Published:** 2026-09-26

**Authors:** Orçun Yalav, Ali Yıldırım, Natavan Guliyeva, İshak Aydın, Serdar Gümüş, Uğur Topal, İsmail Cem Eray, Cem Kaan Parsak

**Affiliations:** Department of General Surgery, Faculty of Medicine, Çukurova University, 01330 Sarıçam, Adana, Türkiye; oyalav@cu.edu.tr (O.Y.); natavanguliyeva@gmail.com (N.G.); drishakaydin@gmail.com (İ.A.); seredargumus@gmail.com (S.G.); sutopal2005@hotmail.com (U.T.); ismailcemeray.gm7@gmail.com (İ.C.E.); cparsak@cu.edu.tr (C.K.P.)

**Keywords:** rectal cancer, watch-and-wait, organ preservation, clinical complete response, circulating tumor DNA, molecular residual disease, total neoadjuvant therapy, mismatch repair deficiency, response assessment, precision oncology

## Abstract

*Background and Objectives*: Watch-and-wait has become an accepted option for selected patients with rectal cancer who achieve a clinical complete response after total neoadjuvant therapy, sparing them total mesorectal excision and the stoma, bowel, urinary and sexual dysfunction that follow it. The decision to omit surgery, however, still rests on anatomical evidence—digital rectal examination, endoscopy and magnetic resonance imaging—which describes what remains visible rather than what remains viable, and local regrowth in about one in four such patients measures that gap. We examined whether, and how, molecular evidence of response should enter that decision. *Materials and Methods*: Narrative review of PubMed/MEDLINE, Europe PMC and ClinicalTrials.gov (final searches 20 September 2026), prioritizing randomized trials, prospective cohorts, registries and meta-analyses; evidence was classified by its relation to the watch-and-wait decision and reported accuracy figures were recalculated with their denominators. *Results*: Direct evidence comes from six small cohorts, four of them retrospective, and accuracy depended on assay, timepoint and outcome. In a 31-patient ultrasensitive sub-study of a randomized trial, detection at restaging predicted neither sustained response nor relapse, and a positive surveillance result was followed by recurrence in fewer than one in four patients (specificity 27.8%, positive predictive value 23.5%); tumor-informed polymerase chain reaction assays were highly specific but detected only 23% to 74% of residual or regrowing tumors. Mismatch repair testing already redirects treatment before surgery is planned. No molecular assay is validated to decide that surgery may be omitted, and none has been compared with carcinoembryonic antigen, endoscopy and magnetic resonance imaging. *Conclusions*: Molecular response should complement, not replace, clinical and radiological assessment. We propose an integrated complete response—concordant anatomical and molecular response, interpreted against baseline tumor biology—as a research framework, whose most defensible first application is to relax surveillance rather than to escalate treatment.

## 1. Introduction

Colorectal cancer remains one of the largest contributors to global cancer incidence and mortality, and its burden has continued to shift toward younger patients and toward health systems least equipped to absorb it [1,2,3]. Rectal cancer accounts for roughly a third of that burden and carries a disproportionate share of its long-term morbidity, because the organ concerned is small, deeply placed, and functionally irreplaceable. Few areas of gastrointestinal oncology have changed as quickly, or as radically, as the management of locally advanced rectal cancer. Within a single professional generation, the operative question has moved from how to resect the rectum to whether the rectum must be resected at all. Total mesorectal excision (TME), combined with neoadjuvant radiotherapy or chemoradiotherapy, drove local recurrence rates into the single digits and became the benchmark against which every subsequent strategy has been measured [4,5,6]. That success came at a price that patients feel for the rest of their lives: permanent or temporary stomas, low anterior resection syndrome, urinary and sexual dysfunction, and a measurable and durable reduction in health-related quality of life after major postoperative complications [7,8].

Against this background, the observation by Habr-Gama and colleagues that a subgroup of patients treated with chemoradiotherapy had no detectable tumor at all, and that many of them remained free of disease without an operation, was genuinely disruptive [9,10]. Two decades later, organ preservation is no longer an isolated experience of a single center. It is embedded in registries, randomized trials, international consensus statements and clinical practice guidelines, and it has been adopted in real-world practice at an accelerating pace [11,12,13,14,15].

What has not changed at the same speed is how we decide who qualifies. A clinical complete response (cCR) is still defined by what the clinician can see and feel: a normal or near-normal digital rectal examination, a flat white scar with telangiectasia at endoscopy, and the absence of residual tumor signal on high-resolution and diffusion-weighted magnetic resonance imaging (MRI) [16,17]. These are morphological criteria applied to a biological question. The mismatch is not a theoretical concern: in the International Watch & Wait Database, the two-year cumulative incidence of local regrowth was 25.2% among 880 patients judged to have a clinical complete response [11], and it is higher still when near-complete responders are included [18,19].

This review addresses one core issue through three linked questions. First, is a clinical complete response really complete? Second, can molecular response—principally, but not exclusively, circulating tumor DNA (ctDNA)—supply the biological evidence that anatomical assessment cannot? Third, if anatomy and biology disagree, which should we trust, and what would a decision framework that integrates both actually look like? Our position is deliberately conservative in the short term and ambitious in the long term: molecular response is not ready to override clinical judgment, but the definition of complete response is ready to be reopened.

One further question has to be settled before the other three can be answered: what outcome is molecular information meant to improve? The evidence reviewed below points in an unexpected direction. Regrowth itself is not the event to be prevented at all costs, because disease-free survival after salvage total mesorectal excision for regrowth was the same as after total mesorectal excision at restaging in the long-term analysis of the OPRA trial (64% in both groups) [18]. The costs of a missed residuum lie elsewhere: in the minority of regrowths that are not salvageable or that accompany distant spread, since registry and institutional data consistently link local regrowth to a higher risk of distant metastasis, whether as its cause or as its marker [20,21]; and in the burden of intensive surveillance that every patient currently carries because residual disease cannot be excluded. We therefore keep three targets separate throughout: relaxing surveillance safely in patients at very low risk, which we regard as the most defensible first application of a highly sensitive assay; earlier detection of distant disease; and avoidance of non-salvageable regrowth. Prevention of regrowth as such is not among them.

## 2. Materials and Methods

This article is a narrative review, reported in accordance with the Scale for the Assessment of Narrative Review Articles (SANRA) [22]. We searched PubMed/MEDLINE and Europe PMC from database inception to 20 September 2026, combining terms for rectal cancer with terms for watch-and-wait, nonoperative management, organ preservation, clinical complete response, total neoadjuvant therapy, circulating tumor DNA, molecular residual disease, mismatch repair deficiency and radiomics, and we searched ClinicalTrials.gov on the same date for registered studies of circulating tumor DNA in this setting [23]. The full search strings, the dates on which they were run and the number of records retrieved by each are given in Supplementary File S1: Table S1 (PubMed/MEDLINE), Table S2 (Europe PMC) and Table S3 (ClinicalTrials.gov). Searches were supplemented by hand-searching the reference lists of retrieved reviews and by targeted searches for the principal randomized trials and international registries in the field. Records were screened by title and abstract, and potentially eligible full texts were assessed by one author (A.Y.); the selection was verified by a second author (O.Y.), and disagreements were resolved by discussion. Because the aim was a conceptual synthesis rather than an exhaustive systematic review, Embase, Scopus, Web of Science and the Cochrane Library were not searched; studies indexed only in those sources may therefore have been missed, and this is acknowledged as a limitation (Section 8).

Priority was given to randomized controlled trials, prospective cohorts, international registry analyses, systematic reviews and meta-analyses; smaller retrospective series were cited only when they addressed a question for which higher-level evidence does not exist. Conference abstracts without a subsequent full publication, and studies confined to metastatic disease, were not considered; several small series of circulating tumor DNA during total neoadjuvant therapy are available only in abstract form and were therefore excluded [24]. Evidence was classified by its relation to the decision under review: direct (studies that included patients managed by, or formally assessed for, nonoperative management), supporting (rectal cancer treated with neoadjuvant therapy and managed by resection), extrapolated (postoperative colorectal cancer, including circulating tumor DNA-guided adjuvant therapy) and contextual (other tumor sites or settings, such as neoadjuvant immunotherapy in colon cancer). Because the aim was to develop and defend a conceptual position rather than to estimate a pooled effect, no protocol was registered, no formal risk-of-bias appraisal was undertaken and no quantitative synthesis was performed; the review is therefore narrative and interpretive by design, and this should be weighed when reading the argument that follows. Where a primary study reported classification performance, or where the underlying counts could be recovered from the published percentages, sample sizes and *p*-values, we recalculated sensitivity, specificity and predictive values with Wilson 95% confidence intervals; reconstructed values are identified as such where they are reported. Ethical approval was not required, since no patient data were generated or analyzed.

## 3. Organ Preservation: How the Paradigm Was Built

The modern organ preservation pathway is a direct consequence of intensified neoadjuvant treatment. Sequencing all systemic therapy before surgery—total neoadjuvant therapy (TNT)—improved compliance with chemotherapy, increased pathological complete response rates and improved disease-related outcomes in the RAPIDO and PRODIGE 23 trials, even though neither was designed as an organ preservation study [25,26,27,28,29,30]. Higher complete response rates mechanically enlarged the pool of candidates for nonoperative management.

The OPRA trial converted that opportunity into evidence. Among 324 patients randomized to induction or consolidation chemotherapy around long-course chemoradiotherapy, five-year disease-free survival was 71% and 69%, respectively, and did not differ between arms; five-year TME-free survival, however, was 39% with induction and 54% with consolidation chemotherapy [12,18]. It is worth being precise about what OPRA randomized: the sequence of chemotherapy and chemoradiotherapy, not watch-and-wait against surgery. Patients with a complete or near-complete clinical response were offered watch-and-wait, and disease-free survival was compared with historical data, so the trial does not provide randomized evidence that watch-and-wait is oncologically equivalent to immediate total mesorectal excision [12,18]. Two findings from the long-term analysis matter more than the headline rates. First, regrowth is an early event: of 81 regrowths, 94% occurred within two years and 99% within three years. Second, and reassuringly, disease-free survival after salvage TME for regrowth was the same as that of patients who underwent TME at restaging (64% in both groups) [18]. Salvage is therefore not a rescue from failure so much as a planned branch of the pathway, and organ preservation strategies that are properly surveilled do not appear to trade survival for function [31,32].

The path between Habr-Gama’s original observation and OPRA was neither short nor smooth, and it is worth recalling because it explains the field’s caution. Early single-center and institutional series established that nonoperative management was feasible and that outcomes could be acceptable, but they were criticized for selection bias and heterogeneous definitions of response [33,34,35]. The Dutch wait-and-see experience introduced protocolized MRI-based selection and structured follow-up, and its long-term extension demonstrated durable organ preservation in a prospectively followed cohort [36,37]. In parallel, the timing-of-response consortium showed that adding consolidation chemotherapy after chemoradiotherapy and lengthening the interval to assessment increased complete response rates, providing the mechanistic argument for the sequencing question that OPRA would later randomize [38,39,40]. A retrospective series of 113 watch-and-wait patients from Memorial Sloan Kettering Cancer Center then quantified, in a modern setting, both the frequency of regrowth (22 of 113 patients) and the fact that regrowths remain resectable: all 22 were salvaged surgically, and the rectum was preserved in 93 of 113 patients (82%) [20]. It is also worth noting what TNT is not: the PROSPECT trial showed that selective omission of radiotherapy is non-inferior in appropriately selected patients, a reminder that intensification is not uniformly desirable and that the field is simultaneously de-escalating and escalating according to risk [41].

Registry data point to the same conclusion. The International Watch & Wait Database, assembling clinical complete responders from many centers, showed that local regrowth is common but overwhelmingly concentrated in the first two years, that it is usually amenable to salvage, and that distant metastasis risk is concentrated in patients who develop regrowth rather than in the watch-and-wait population as a whole [11,21,42]. Pooled analyses and propensity-matched cohorts have generally found comparable survival between watch-and-wait and immediate surgery in appropriately selected patients, while quality-of-life comparisons favor organ preservation [8,43,44,45,46,47,48]. These estimates are not interchangeable because the populations differ: the registry analysis was restricted to patients with a clinical complete response [11], the individual participant data meta-analysis to studies using criteria equivalent to the São Paulo benchmarks [42], whereas OPRA also offered watch-and-wait to near-complete responders [18,19]. Table 1 summarizes the principal trials, registries and pooled analyses, with what each can and cannot show.

The strategy has therefore matured from an anecdote into a defensible standard of care for selected patients [14,54,55,56,57]; Figure 1 places this second era between radical surgery for all patients and the proposed, still investigational, third era discussed in Section 7. What remains unresolved is the selection step itself, and this is where the central conflict of the field now sits: how much oncological uncertainty are we prepared to accept in order to preserve the rectum?

## 4. Is Clinical Complete Response Really Complete?

Contemporary response assessment rests on three pillars—digital rectal examination, endoscopy, and high-resolution MRI supplemented by diffusion-weighted imaging—used together rather than in isolation [16,17,58,59]. Each pillar is informative and each has a defined failure mode (Table 2).

### 4.1. Digital Rectal Examination and Endoscopy

Digital rectal examination is inexpensive, immediate, and irreplaceable for low tumors, but it is operator-dependent and, as recent work on standardized reporting emphasizes, is rarely documented in a way that permits comparison between examiners or over time [60]. Endoscopy visualizes the mucosa with high fidelity and has generated the descriptive vocabulary of the clinical complete response—the whitened scar, telangiectasia, the absence of ulceration—yet the mucosa is the one compartment in which residual disease is least likely to hide [16,61]. Submucosal or intramural nests, and mesorectal nodal deposits, are invisible to it. The call to standardize endoscopic documentation in watch-and-wait programs reflects how much clinical weight is being placed on an assessment that has never been formally standardized [62].

### 4.2. Magnetic Resonance Imaging

MRI is the most quantitative of the three and the most frequently over-interpreted. Its central problem after chemoradiotherapy is that fibrosis and viable tumor share a low-signal appearance on T2-weighted sequences, so that the same dark scar may represent cure or persistence [58,63,64]. Diffusion-weighted imaging improves discrimination and has become integral to restaging, but its interpretation is not free of subjectivity, and reader agreement in multireader studies is only moderate [65,66,67,68]. Restaging MRI performs considerably better at excluding gross residual disease than at proving the absence of microscopic residual disease, and the mesorectal nodal compartment remains a particular blind spot: nodal disease can persist in patients whose primary tumor bed appears completely healed at endoscopy [63,69].

A structural difficulty underlies all three modalities: rectal cancer after chemoradiotherapy does not behave like the measurable lesions for which conventional response criteria were designed. The Response Evaluation Criteria in Solid Tumors (RECIST) were designed to capture changes in the longest diameter of a measurable mass and are largely uninformative for a tumor that has been replaced by scar tissue of similar dimension [70]. Magnetic resonance tumor regression grading was developed precisely to fill that gap and has been evaluated prospectively as a stratification biomarker, but the same interpretive uncertainty that limits T2-weighted assessment limits the grading system built upon it [58,71]. The consequence is that the field has accumulated a rich descriptive vocabulary for what a responding rectal tumor looks like without acquiring a corresponding measure of what it contains [72].

### 4.3. Timing, Near-Complete Response and the Regrowth Signal

Timing compounds the problem. Response is a continuous process rather than a fixed endpoint; assessments performed too early misclassify slow responders as incomplete, and the “near-complete” category—small residual abnormality that may either resolve or progress—occupies an uncomfortable middle ground with its own management logic and prognosis [19,40,73]. In the OPRA secondary analysis, outcomes differed systematically across clinical response grades: among patients who entered watch-and-wait, local regrowth occurred in 27 of 123 (22.0%) with a clinical complete response and in 48 of 94 (51.1%) with a near-complete response, and three-year organ preservation was 77% and 40%, respectively [19]. Response is a gradient that we currently force into a binary decision, and regrowth rates from cohorts that admitted near-complete responders are therefore not comparable with those restricted to strict complete responders.

The most direct evidence that cCR is an imperfect surrogate comes from the outcome itself. If clinical complete response were biologically complete, regrowth would be rare. In registry and pooled data restricted to clinical complete responders, the two-year cumulative incidence of regrowth is close to one in four—25.2% among 880 patients in the International Watch & Wait Database [11] and 21.4% among 602 patients in an individual participant data meta-analysis [42]—and it is roughly twice as high after a near-complete response [19]. Concordance between clinical and pathological complete response is likewise imperfect in both directions: some patients judged incomplete are found to have no viable tumor at surgery, while others judged complete harbor residual disease [32,74,75,76]. The inescapable conclusion is that we are using anatomical tools to answer a biological question. We do not lack precision in describing the tumor bed; we lack a measure of whether the tumor still exists.

## 5. Molecular Response: The Missing Layer

### 5.1. What Molecular Response Measures

Molecular response is a different construct from radiological or clinical response. It does not ask whether the tumor has shrunk or whether the mucosa has healed; it asks whether tumor-derived biological signal is still detectable. The candidate analytes are several—circulating tumor cells, circulating RNA species, methylation signatures, and fragmentomic patterns—but ctDNA is the only one with sufficient maturity to be discussed as a decision aid in this setting [77,78,79,80,81,82].

The rationale is straightforward. Tumor cells shed fragmented DNA carrying somatic variants into plasma; when the tumor is eradicated, the signal disappears with a half-life measured in hours rather than months, so ctDNA reflects current disease burden rather than a historical anatomical footprint [83,84,85]. In colorectal cancer, ctDNA detected after curative resection identifies molecular residual disease and predicts recurrence with a magnitude of effect that dwarfs conventional clinicopathological factors, an observation replicated from the earliest stage II cohorts through to large prospective registries [86,87,88,89,90,91]. That evidence, however, is extrapolated rather than direct: it was generated after the tumor had been removed. The DYNAMIC trial went further and demonstrated that a ctDNA-guided strategy could reduce adjuvant chemotherapy use without compromising recurrence-free survival, establishing, in the adjuvant setting, the principle that ctDNA can change management rather than merely describe risk [92].

Transposing this logic to organ preservation is intuitive but not trivial. In the adjuvant setting, the tumor has already been removed and ctDNA asks whether anything was left behind. In watch-and-wait, the tumor bed is still in situ and ctDNA is asked a harder question: whether an unresected, irradiated, fibrotic lesion still contains viable malignant cells. The relevant question for this review is therefore narrower than the general enthusiasm for liquid biopsy: could molecular negativity provide the reassurance that anatomical assessment cannot, and could molecular positivity identify the patient whose apparently perfect response is an illusion?

### 5.2. Circulating Tumor DNA as a Molecular Safety Net

It is useful to separate three distinct windows in which ctDNA might contribute [93]. The primary studies that inform these windows in patients considered for watch-and-wait are few and small; they are summarized in Table 3, and their classification performance, where it can be derived, in Table 4.

Before treatment, baseline ctDNA characterizes shedding and disease burden. Its main value is technical rather than clinical: a tumor that does not shed measurably at baseline cannot be monitored meaningfully afterwards, so baseline sampling defines who is informative for later testing. In a tumor-informed whole-genome sequencing analysis of 31 patients enrolled in OPRA at a single institution, ctDNA was detectable at baseline in 20 of the 21 patients with a baseline sample (95%), with a median tumor fraction of only 0.081%, and a lower baseline tumor fraction was associated with a higher probability of sustained clinical complete response [94]. Baseline burden therefore behaves as a prognostic covariate in its own right.

During and immediately after TNT, ctDNA measures the trajectory of response rather than its endpoint, and the mid-treatment and restaging timepoints of the OPRA sub-study gave discordant results. Midway through treatment, after completion of the first component of TNT, ctDNA was detected in 12 of 28 patients; a sustained clinical complete response followed in 16.7% of those with detectable ctDNA and in 75.0% of those without (*p* = 0.006), and detection carried a hazard ratio (HR) for relapse of 6.47 (95% confidence interval [CI] 1.33–31.41) [94]. At restaging 8 ± 4 weeks after the end of TNT—the moment at which the watch-and-wait decision is actually made—ctDNA was detected in 17 of 26 patients, and detection was associated with neither a sustained clinical complete response among watch-and-wait candidates (71.4% versus 60.0%; *p* = 1.000) nor relapse (HR 1.63, 95% CI 0.61–8.07) [94]. An interval as wide as 1.33 to 31.41 is compatible with a modest association and with an extreme one, and the null result at restaging rests on equally few events. The investigators themselves described the discordance as counterintuitive: 12 of the 15 patients without detectable ctDNA at the earlier timepoint became positive at restaging, only two of them relapsed, and their baseline tumor fractions were lower, which they interpreted as a possible loss of specificity in low-shedding tumors [94].

Before entering watch-and-wait, ctDNA is asked to interrogate molecular residual disease at the moment of decision, and the evidence here comes from small cohorts that used different assays and different definitions of positivity. In a single-institution retrospective case series of 28 patients treated between 2019 and 2023—cited because no higher-level evidence addresses this question—surgery for the rectal primary was required in 6 of 9 ctDNA-positive patients (66.7%, 95% CI 35.4–87.9%) and in 4 of 19 ctDNA-negative patients (21.1%, 95% CI 8.5–43.3%; *p* = 0.035) [95]. The intervals overlap; positivity was defined by any positive result during post-treatment follow-up rather than at a prespecified timepoint, and only 4 of the 9 patients with residual cancer in the resected specimen had ctDNA detected before surgery. The proportion reported as sustaining nonoperative management in the ctDNA-negative group (74%) does not reconcile with the 15 of 19 patients who did not undergo surgery, and the outcome of the remaining patient is not stated in the source; we therefore report surgery for the rectal primary as a single outcome definition for both groups. In the NOMINATE trial, a prospective biomarker study of 64 patients using a tumor-informed polymerase chain reaction assay, ctDNA detected at final restaging had a specificity and a positive predictive value of 100% for pathological residual disease, but its sensitivity was modest, and ctDNA was detected during surveillance in only two of the five patients whose tumor regrew [96]. In a retrospective real-world cohort, 14 of 64 patients managed nonoperatively had ctDNA detected at some point after neoadjuvant therapy and all 14 relapsed, against 5 of 50 patients who remained ctDNA-negative [97]; in a single-center retrospective cohort, by contrast, post-treatment ctDNA identified only 23% of the residual tumors found at resection, with a negative predictive value of 47% [98]. Consistency of direction across cohorts of this size is weak support for a clinical construct.

A systematic review and meta-analysis of 22 studies and 1676 patients with locally advanced rectal cancer reported pooled hazard ratios for recurrence of 8.87 (95% CI 4.91–16.03) for ctDNA detected after neoadjuvant treatment and 15.15 (95% CI 8.21–27.95) for ctDNA detected after surgery [99]. These are two separate pooled estimates, derived from the subsets of the eight studies that contributed quantitative data at each timepoint rather than from a within-patient progression, and they come predominantly from patients who proceeded to resection. A prognostic estimate obtained in a surgically managed population does not translate directly to the decision considered here, which concerns patients in whom surgery may be omitted.

The pooled hazard ratio of 8.87 and the null result at restaging in the OPRA sub-study appear to contradict one another, and the contradiction is instructive. The most plausible explanation is analytical: an ultrasensitive whole-genome assay detects low-tumor-fraction signal far more often than the targeted and tumor-agnostic assays pooled in the meta-analysis, and therefore detects far more signal in patients who are cured, buying sensitivity at the cost of specificity [94,99]. The polymerase chain reaction-based tumor-informed studies show the mirror image, with specificity and positive predictive values approaching 100% and modest sensitivity [96,98]. Differences in population, in the timepoint sampled and in the outcome defined, together with few events and the publication bias that the meta-analysis itself acknowledges, account for the rest [99]. The practical conclusion is that the performance of ctDNA in this setting is a property of a particular assay at a particular timepoint in a particular population, and not of the analyte.

**Table 3:** Primary studies of circulating tumor DNA after neoadjuvant therapy that inform the organ-preservation decision.

Study and Design	Patients (Events)	ctDNA Assay	Timepoints Sampled	Principal Finding for the Organ-Preservation Decision	Evidence
Manca et al. [94]; analysis of prospectively collected samples from a single institution within the OPRA randomized trial	31 (10 recurrences; 6 regrowths among 24 offered watch-and-wait)	Tumor-informed whole-genome sequencing; limit of detection not stated	Baseline (n = 21); during TNT (n = 28); restaging 8 ± 4 weeks after TNT (n = 26); surveillance (22 patients, 102 samples)	Detection during TNT was associated with a lower rate of sustained clinical complete response and with relapse; detection at restaging was associated with neither; surveillance positivity was 100% sensitive but 27.8% specific for recurrence	Direct
Akiyoshi et al. [96]; biomarker study within the randomized phase II NOMINATE trial (multicenter)	64 (25 managed nonoperatively; 5 local regrowths)	Tumor-informed multiplex PCR–NGS (Signatera)	Baseline; two interim timepoints; final restaging; after surgery or during nonoperative management (412 samples)	Detection at restaging was 100% specific with a positive predictive value of 100% for pathological residual disease, but sensitivity was modest; ctDNA was detected during surveillance in 2 of 5 regrowths	Direct
Kim et al. [95]; single-institution retrospective case series	28 (10 operated for suspected regrowth; 3 distant recurrences)	Tumor-informed multiplex PCR–NGS (Signatera); reported analytical sensitivity > 95% at 0.01% mean variant allele frequency	Serial after TNT (≥4 weeks after treatment and within 2 weeks of restaging) and during follow-up	Surgery for the rectal primary in 6 of 9 ctDNA-positive versus 4 of 19 ctDNA-negative patients (*p* = 0.035); ctDNA detected before surgery in 4 of 9 patients with residual cancer	Direct
Chakrabarti et al. [97]; retrospective real-world cohort of commercially ordered tests	220 (72 managed nonoperatively, 64 with post-treatment ctDNA; 19 relapses)	Tumor-informed 16-plex PCR–NGS (Signatera)	Serial after neoadjuvant therapy (first sample at a median of 2.9 months)	Relapse in 14 of 14 ctDNA-positive versus 5 of 50 ctDNA-negative patients managed nonoperatively; 13 of the 14 relapses were local	Direct
Alden et al. [98]; single-center retrospective cohort	44 (22 resected, of whom 13 had residual disease)	Tumor-informed multiplex PCR–NGS (Signatera)	Before, during and after TNT (median 1.1 weeks after completion) and after surgery	Post-TNT ctDNA was 23% sensitive and 100% specific for residual disease at resection, with a negative predictive value of 47%	Direct
Hulse et al. [100]; single-institution retrospective surveillance series	144 tested; 28 recurrences (8 during watch-and-wait)	Commercial ctDNA testing	During post-treatment surveillance	ctDNA was positive before clinical detection in 20 of 28 recurrences (median 100 days), in 11 of 12 distant but only 10 of 17 local recurrences	Direct
Vidal et al. [101]; biomarker study within the randomized phase II GEMCAD 1402 trial	72 (all resected)	Plasma-only assay integrating genomic and epigenomic signatures	Baseline and within 48 h before surgery after TNT	ctDNA detected before surgery in 15%; not associated with pathological response but associated with systemic recurrence (disease-free survival HR 4.0)	Supporting
O’Sullivan et al. [99]; systematic review and meta-analysis	1676 patients in 22 studies (8 in the quantitative synthesis)	Mixed (11 tumor-agnostic, 10 targeted, 1 both)	After neoadjuvant therapy; after surgery	Pooled hazard ratios for recurrence of 8.87 after neoadjuvant therapy and 15.15 after surgery, from different subsets of studies in predominantly resected populations	Supporting

Direct evidence: studies including patients managed by, or formally assessed for, nonoperative management. Supporting evidence: rectal cancer treated with neoadjuvant therapy and managed by resection. ctDNA: circulating tumor DNA; TNT: total neoadjuvant therapy; PCR–NGS: polymerase chain reaction–next-generation sequencing; HR: hazard ratio; OPRA, NOMINATE and GEMCAD 1402 are trial acronyms.

**Table 4:** Classification performance of circulating tumor DNA at defined timepoints, with the prevalence of the outcome in the population tested.

Study and Timepoint	Outcome (Prevalence in the Population Tested)	Sensitivity (95% CI)	Specificity (95% CI)	Positive Predictive Value (95% CI)	Negative Predictive Value (95% CI)
Manca et al. [94], during TNT	Failure to achieve a sustained clinical complete response (14 of 28; 50.0%)	71.4% (45.4–88.3) †	85.7% (60.1–96.0) †	83.3% (55.2–95.3) †	75.0% (50.5–89.8) †
Manca et al. [94], restaging after TNT	Sustained clinical complete response in watch-and-wait candidates (counts not reported) ‡	—	—	—	—
Manca et al. [94], any surveillance timepoint	Disease recurrence (4 of 22; 18.2%)	100% (51.0–100) †	27.8% (12.5–50.9) †	23.5% (9.6–47.3) †	100% (56.6–100) †
Akiyoshi et al. [96], restaging after TNT	Pathological residual disease in resected patients (counts not reported)	Modest (not quantified)	100%	100%	—
Akiyoshi et al. [96], surveillance	Local regrowth (5 events)	40.0% (11.8–76.9) †	—	—	—
Kim et al. [95], any timepoint after TNT	Surgery for the rectal primary (10 of 28; 35.7%)	60.0% (31.3–83.2) †	83.3% (60.8–94.2) †	66.7% (35.4–87.9) †	78.9% (56.7–91.5) †
Chakrabarti et al. [97], serial after neoadjuvant therapy	Relapse during nonoperative management (19 of 64; 29.7%)	73.7% (51.2–88.2) †	100% (92.1–100) †	100% (78.5–100) †	90.0% (78.6–95.7) †
Alden et al. [98], after TNT	Residual disease at resection (13 of 22; 59.1%)	23.1% (8.2–50.3) †	100% (70.1–100) †	100% (43.9–100) †	47.4% (27.3–68.3) †
Hulse et al. [100], surveillance	Recurrence (28 events; patients without recurrence not analyzed)	71.4% (52.9–84.7) †	—	—	—
O’Sullivan et al. [99], after neoadjuvant therapy and after surgery	Recurrence (pooled; hazard ratios only)	—	—	—	—

† Calculated by the present authors from the counts reported in the source or reconstructed from the published percentages, sample sizes and *p*-values; Wilson 95% confidence intervals. ‡ At restaging, detection was associated with neither a sustained clinical complete response (71.4% versus 60.0%; *p* = 1.000) nor relapse (hazard ratio 1.63, 95% CI 0.61–8.07). —: could not be derived from the published report. Predictive values depend on the prevalence shown and cannot be transported to populations with a different prevalence. TNT: total neoadjuvant therapy; CI: confidence interval.

A practical caveat cuts across all three windows. Tumor-informed assays, which build a personalized panel from sequenced tumor tissue and then track those variants in plasma, achieve the highest analytical sensitivity and are the platform on which most of the rectal data above were generated; they require adequate tumor tissue, add turnaround time, and are more expensive. Tumor-agnostic or plasma-only assays avoid the tissue requirement and can be deployed faster, at the cost of lower sensitivity for low-burden disease and greater vulnerability to non-tumor signal [81,88,91]. In an organ preservation pathway where the decision window is short and the residual burden is by definition minimal, this trade-off is not academic: the assay chosen largely determines what question can be answered. Analytical and clinical sensitivity should not be confused. An assay validated to detect variants at a mean allele fraction of 0.01% under controlled conditions [95] may still miss residual tumor in practice—in the studies above, between 23% and 74% of residual or regrowing tumors were detected [95,96,97,98]—because clinical sensitivity also depends on how much DNA a small, irradiated and often fibrotic residuum sheds, on plasma volume and sampling time, and on the number of variants tracked.

Hazard ratios of this size explain the enthusiasm, but they cannot support a decision rule. They are prognostic associations measured in cohorts in which ctDNA did not influence management, and they say nothing directly about how often a given result would be correct in an individual patient. What a clinician needs is sensitivity, specificity and predictive values at a defined timepoint, together with the prevalence of residual disease in the population tested; Table 4 assembles these wherever they can be derived, and its empty cells are themselves a finding. The clinically decisive scenarios are the two that no dataset has yet resolved. The first is the patient with a negative digital rectal examination, negative endoscopy, negative MRI and undetectable ctDNA—the lowest apparent residual-risk phenotype, and a plausible ideal candidate for nonoperative management. The second is the patient with the same negative anatomical assessment but persistent ctDNA positivity. Should that patient be operated on? At present, the honest answer is that we do not know, and the burden of proof for recommending an unnecessary proctectomy on the strength of a plasma assay is properly a high one.

### 5.3. The Discordant Response Problem

The interesting cases are the discordant ones, and they are best organized as four clinical–molecular phenotypes (Table 5). The two axes of that table are the two response layers of the integrated complete response proposed in Section 7.1: anatomical response, assessed clinically and radiologically, and molecular response. A clinical complete response with negative molecular testing represents the deepest apparent response and the phenotype in which watch-and-wait is most defensible. A clinical complete response with positive molecular testing suggests occult residual disease, but may equally reflect assay noise, clonal hematopoiesis, or shedding from a subclinical distant focus that surgery of the rectum would not address. How often such a result is a true positive depends on the assay and on the prevalence of residual disease: with the ultrasensitive whole-genome assay, a positive result at any surveillance timepoint was followed by recurrence in fewer than one in four patients (positive predictive value 23.5%, 95% CI 9.6–47.3%) [94], whereas with polymerase chain reaction-based tumor-informed assays the positive predictive value ranged from 66.7% to 100% [95,96,97,98]. An incomplete clinical response with negative molecular testing may indicate a genuinely low-shedding tumor, a delayed responder, or residual fibrosis misread as tumor. An incomplete clinical response with positive molecular testing is the least ambiguous combination and points to persistent disease requiring standard oncological management.

Two asymmetries deserve emphasis. First, the sensitivity of ctDNA in localized rectal cancer is fundamentally constrained by shedding biology. Small-volume, well-differentiated, luminal residual disease may release too little DNA to be detected by any assay, so a negative result excludes far less than clinicians intuitively assume [79,91]; how much less—that is, the negative predictive value—depends in addition on the prevalence of residual disease in the population tested, which differs substantially between complete and near-complete responders [19]. Second, ctDNA reports systemic tumor burden without localizing it, so its specificity for local residual disease is limited: a positive result in a patient with a pristine rectum may be the earliest sign of distant rather than local failure—the pattern suggested by registry data linking distant metastasis risk to regrowth [21] and by the preferential detection of distant over local recurrences in a surveillance series [100]. The positive predictive value that follows from these properties is, again, a function of prevalence and cannot be transported from one population to another.

The question “which response should we trust when anatomy and biology disagree?” therefore has no single answer. It has a decision-analytic answer: the two modalities carry different error structures, and their combination is more informative than either alone, but only if the combination is prespecified rather than improvised at the multidisciplinary meeting. Until prospective trials define what action follows each phenotype, these categories are conceptual scaffolding and must not be used as standalone treatment algorithms. In practice, the first response to molecular positivity in a patient with an apparently healed rectum should be complete local and systemic restaging, not a decision about proctectomy.

## 6. Beyond Circulating Tumor DNA: Tumor Biology and Immunotherapy

### 6.1. Biomarkers of Organ Preservation

Molecular response is not synonymous with ctDNA. Several other layers of biology plausibly influence whether an organ preservation attempt will succeed, and they differ in how close they are to clinical use.

Mismatch repair status is the clearest example of a biomarker that has already changed the treatment offered rather than merely predicting outcome, and it is discussed separately below. Tumor genomics—*KRAS*, *NRAS*, *BRAF*, *TP53*, *APC*, *SMAD4* and *HER2* alterations, and the broader consensus molecular subtypes—carry prognostic weight in colorectal cancer generally, but evidence that any single alteration should modify a watch-and-wait decision remains insufficient [82,102,103]. It is worth stating plainly that a biomarker can be strongly prognostic and still be useless for this specific decision, because the decision requires discrimination at the level of the individual tumor bed rather than at the level of the cohort.

The tumor immune microenvironment is conceptually attractive because radiotherapy and chemoradiotherapy produce part of their effect through immunogenic mechanisms. CD8-positive T-cell infiltration, tumor-infiltrating lymphocyte density and composite immune signatures have been associated with pathological response to neoadjuvant treatment, and immune-related transcriptomic models have been constructed for locally advanced rectal cancer, but these remain research instruments with no external validation adequate for clinical deployment [103,104,105].

Imaging-derived quantitative features occupy a middle position. Radiomic and artificial-intelligence models applied to restaging MRI have shown reproducible signal for predicting pathological complete response, and a systematic review and meta-analysis of such models reported clinically interesting discrimination, yet performance degrades on external validation and no model has been shown to improve patient selection prospectively [64,76,106]. The most credible future for this line of work is not as a competitor to ctDNA but as a complementary modality: imaging localizes, molecular testing quantifies burden, and multimodal models that combine MRI features, pathology, ctDNA and genomics are the natural next step [102,107].

### 6.2. Immunotherapy Is Already Redefining Organ Preservation

The strongest existing proof that molecular information can reshape the decision to operate comes not from ctDNA but from mismatch repair testing. In mismatch repair-deficient locally advanced rectal cancer, single-agent programmed cell death protein 1 blockade with dostarlimab produced a clinical complete response in all 12 patients of the initial cohort (95% CI 74–100%), none of whom had received chemoradiotherapy or undergone surgery at the time of the report [52]. In the expanded experience, all 49 patients of the rectal cohort who completed treatment achieved a clinical complete response and elected nonoperative management, and 37 had a sustained clinical complete response at 12 months, which met the prespecified efficacy criterion [53]; an interim analysis of one or two cycles of nivolumab plus ipilimumab reported a clinical complete response in all 16 patients treated for mismatch repair-deficient rectal cancer [108]. In locally advanced mismatch repair-deficient colon cancer, neoadjuvant nivolumab plus ipilimumab produced a pathological response in 109 of 111 patients, including 75 (68%) pathological complete responses [109], consistent with the earlier pilot experience [110]. These colon data are contextual rather than direct evidence for the rectum: they establish the biological sensitivity of mismatch repair-deficient tumors to checkpoint blockade, not the safety of watch-and-wait after it.

The conceptual significance of these results is larger than their epidemiological footprint, since mismatch repair deficiency accounts for only a small minority of rectal cancers. For that minority, a molecular test performed before any treatment determines which treatment is given—checkpoint blockade rather than chemoradiotherapy—and thereby opens the possibility that surgery is never needed; whether surgery is actually omitted still depends on achieving a clinical complete response and sustaining it under intensive surveillance [53]. That remains a qualitative change in what a biomarker is allowed to decide. Efforts to extend immunotherapy to mismatch repair-proficient, microsatellite stable disease are more tentative: in the mismatch repair-proficient cohort of the NICHE study, neoadjuvant nivolumab plus ipilimumab produced responses in 26% of 31 patients with early-stage colon cancer, including six major pathological responses [111], and watch-and-wait after immunotherapy-containing neoadjuvant treatment of mismatch repair-proficient rectal cancer has so far been described only in real-world observational series [112]. The boundary of biomarker-directed organ preservation is being explored rather than moved.

If a single molecular test can already redirect one subgroup onto a pathway in which proctectomy is frequently avoided, the argument that molecular data have no place in the response assessment of other subgroups becomes difficult to sustain. The question is no longer whether biology may inform the decision to omit surgery, but which biology, measured when, and with what evidentiary threshold.

## 7. Toward an Integrated Complete Response and Precision Watch-and-Wait

### 7.1. Should Complete Response Be Redefined?

Our central proposal is definitional rather than therapeutic. Complete response is currently a single-layer concept assessed by clinical and radiological means. We suggest that it be reconceived as a two-layer construct, interpreted against the baseline biology of the tumor.

The first layer is anatomical response: digital rectal examination, endoscopy, and high-resolution and diffusion-weighted MRI, assessed and documented in a standardized manner—in other words, the clinical and radiological assessment that currently defines a clinical complete response [17,58,59,60,62]. The second layer is molecular response: the presence or absence of detectable molecular residual disease, principally by tumor-informed ctDNA, measured serially rather than at a single timepoint [94,95,99]. We define an integrated complete response (iCR) as a concordant complete response in both layers—an anatomical clinical complete response together with a negative molecular assessment in a patient whose tumor was shown to shed ctDNA at baseline (Figure 2). Mismatch repair and microsatellite status, the somatic alterations discussed in Section 6.1 and the immune phenotype do not constitute a third response layer: they are intrinsic baseline features of the tumor, they do not change with treatment, and they therefore cannot be concordant or discordant with a response. We treat them instead as baseline biological context, which modifies the expected response trajectory and the interpretation of both layers [52,102,110]. Table 6 proposes an operational specification for each layer, including the handling of an uninformative molecular result.

We advance iCR explicitly as a research construct and a common vocabulary for trial design, not as a new clinical criterion. It has three practical merits. It makes discordance visible and therefore reportable, rather than allowing it to be resolved silently by whichever modality the individual clinician trusts most. It creates a natural stratification variable for prospective studies, which currently randomize or observe patients defined only by anatomical response. And it prevents the field from making the opposite error to the one it made with imaging: replacing an imperfect anatomical gold standard with an imperfect molecular one.

### 7.2. From Watch-and-Wait to Precision Watch-and-Wait

The current model is linear: clinical complete response leads to watch-and-wait. A precision model would be multidimensional, combining anatomical response, molecular response, baseline tumor biology, and—decisively—the individual patient’s tolerance of risk (Figure 1).

That last element is frequently omitted from biomarker discussions and should not be. Watch-and-wait is one of the few oncological decisions in which the trade-off is not between survival and survival but between function and uncertainty. Patients differ in how they weigh a stoma against a probability of regrowth, and quality-of-life data comparing organ preservation with resection consistently show that the difference matters to them [7,8,48]. A patient with a strong preference for rectal preservation and a demonstrable willingness to attend intensive surveillance may reasonably accept a risk profile that another patient would decline. Molecular data do not remove this negotiation; they make it better informed by narrowing the width of the uncertainty being discussed. The negotiation is also not identical across patients: in early-onset rectal cancer, where the functional consequences of proctectomy are measured over decades and fertility and sexual function weigh heavily, the calculus differs from that of an older patient with competing comorbidity [113,114].

What might this look like operationally? A plausible, testable framework would stratify patients with an apparent clinical complete or near-complete response into three tiers. In the first, anatomical and molecular assessments agree and are both negative; surveillance follows the standard schedule and de-escalation could be studied. In the second, anatomical assessment is complete but molecular testing is positive or equivocal; complete local and systemic restaging is performed, surveillance is intensified, the interval to the next MRI and endoscopy is shortened, and the patient is explicitly counseled that the residual-risk estimate is higher than the imaging alone suggests. In the third, anatomical assessment is near-complete; the decision is deferred and reassessment is scheduled within a short interval [19,31,40]. Local excision of a small residual abnormality is sometimes proposed in this tier as a diagnostic as well as a therapeutic step, and it deserves an explicit caveat. The only randomized evidence, GRECCAR 2, was confined to small cT2–T3 low rectal cancers of no more than 4 cm with a residual lesion of no more than 2 cm after chemoradiotherapy; superiority of local excision over total mesorectal excision was not shown, largely because 26 of 74 patients in the local excision arm required completion total mesorectal excision for ypT2–T3 disease, which added morbidity [49], although five-year local recurrence, metastasis and survival did not differ between arms [50]. Local excision samples the bowel wall only and leaves the mesorectal nodes, in which disease can persist beneath a healed mucosa [69], unassessed. Nothing in this framework requires molecular testing to overrule clinical judgment; it requires only that molecular information be allowed to modulate the intensity of what follows.

Watch-and-wait should therefore evolve from a response-based strategy into a multidimensional, risk-adapted strategy in which the intensity of surveillance, the threshold for salvage, and the counseling offered are all calibrated to an individually estimated risk of residual disease rather than to a binary label. Whether such calibration improves outcomes is an empirical question, and it is the question that the next generation of trials should be built to answer [13,107].

### 7.3. Molecular Surveillance After Entering Watch-and-Wait

Molecular testing need not be confined to selection. Surveillance after entering watch-and-wait is currently intensive and almost entirely anatomical: serial digital rectal examination, endoscopy and MRI at short intervals during the first two years, with carcinoembryonic antigen measurement and cross-sectional imaging for distant disease [14,57,115,116]. The intensity is justified by the timing of regrowth, which is overwhelmingly concentrated in the first two years [11,18,42]. Guideline recommendations for this population are, understandably, based more on expert consensus than on comparative evidence, and they differ between societies in both interval and modality [14,57,115,116]. The evidence base for surveillance intensity in resected colorectal cancer, by contrast, is notably unimpressive, with randomized trials failing to demonstrate a survival advantage for more frequent testing [117,118,119].

Serial ctDNA could plausibly change this in either of two directions. If molecular recurrence reliably precedes radiological regrowth, a positive result could trigger intensified imaging and earlier salvage, converting a fixed surveillance schedule into an adaptive one. If, conversely, sustained molecular negativity proves sufficiently reliable in excluding regrowth and distant disease in this specific setting—so that, at the prevalence of residual disease seen after a clinical complete response, a negative result carries a high negative predictive value—it could permit de-escalation of a surveillance program that is burdensome, expensive and anxiety-provoking. The former possibility is the one most often invoked; the latter may be the more valuable, and both require the same prospective data to establish.

Either direction has to be demonstrated against the existing combination of carcinoembryonic antigen, endoscopy and MRI, rather than against no testing at all. To our knowledge, that comparison has not been made in patients managed by watch-and-wait: none of the primary studies in Table 3 compared ctDNA with carcinoembryonic antigen, and in the OPRA sub-study tumor fraction and carcinoembryonic antigen were observed to fluctuate in parallel in selected patients, without any formal comparison [94]. In resected colorectal cancer, where the question has been asked directly, a tumor-informed assay identified 8 of 15 recurrences during surveillance, against 11 of 15 for imaging combined with carcinoembryonic antigen, with no difference in the time to detection [120]. The incremental value of a molecular test over the tests already in use is therefore an open question in both settings, and it should be a prespecified endpoint of the trials proposed in Section 9.

The critical unknown is lead time. Evidence that ctDNA detects molecular residual disease months before clinical recurrence in the postoperative colorectal setting is reasonably strong [86,87,89,90]. For local regrowth in an unresected, irradiated rectum, where the tumor volume at the moment of detectable regrowth may be smaller than the assay’s effective threshold, the data are few and inconsistent. In a retrospective surveillance series of 28 recurrences, 8 of which occurred during watch-and-wait, ctDNA became positive before clinical detection in 20, at a median of 100 days, but in 11 of 12 distant recurrences and in only 10 of 17 local recurrences [100]; in the NOMINATE trial, ctDNA was detected during surveillance in two of the five patients whose tumor regrew [96]; and in a real-world cohort, all six patients who converted from negative to positive during serial testing subsequently developed local recurrence [97]. Until lead time is measured prospectively and with a defined sampling schedule, ctDNA surveillance in watch-and-wait should be regarded as a research procedure performed alongside—never instead of—endoscopic and MRI follow-up.

## 8. Limitations: The Dangers of Moving Too Fast

Enthusiasm for molecular response assessment must be balanced against a set of limitations that are neither trivial nor close to resolution.

Shedding is the first and most fundamental. Localized rectal cancer sheds far less DNA than metastatic disease, and after chemoradiotherapy the residual burden is smaller still. The tumor fractions being measured in this setting are extraordinarily low—a median of 0.081% among the 21 baseline samples of the OPRA sub-study, before any treatment effect [94]. Assay sensitivity, not biology, may therefore determine the result.

Specificity is the mirror problem, and it is currently the more troubling one. The null association at restaging reported in Section 5.2 is best read as a failure of specificity rather than as evidence that post-treatment ctDNA is uninformative: in the same ultrasensitive analysis, ctDNA was detected at restaging in 17 of 26 patients, and across surveillance timepoints sensitivity for recurrence was 100% but specificity was 27.8% and the positive predictive value 23.5% [94]. An assay that flags most patients, including most of those who are cured, cannot be used to send patients to the operating room; the investigators concluded that this pattern “poses some concerns for the clinical implementation of ultra-sensitive ctDNA assays” in this setting [94]. The polymerase chain reaction-based studies show the opposite profile, with high specificity and modest sensitivity [96,98], so that the choice of assay determines which of the two errors the clinician accepts.

Several further constraints apply. Tumor-informed assays require adequate tissue and a turnaround time that may not fit the restaging window, while tumor-agnostic assays trade that constraint for lower sensitivity [88,91]. Clonal hematopoiesis and other non-tumor sources of variant signal generate false positives that require paired leukocyte sequencing to exclude [79]. Sampling timepoints, plasma volumes, variant-calling thresholds and definitions of positivity vary between studies, so that pooled effect estimates conceal substantial methodological heterogeneity [91,99]. Cost and access are not negligible considerations for health systems already struggling to fund the MRI capacity that watch-and-wait requires. And the interpretation of molecular positivity in a clinically negative patient—the phenotype that motivated this article—has no evidence-based management at all.

This review has limitations of its own. It is narrative rather than systematic; the search was confined to PubMed/MEDLINE, Europe PMC and ClinicalTrials.gov, so that studies indexed only in Embase, Scopus, Web of Science or the Cochrane Library may have been missed; records were screened by one author and the selection verified by a second, rather than in duplicate; conference abstracts were excluded, which removed several small ctDNA series reported only in abstract form [24]; and the classification figures in Table 4 inherit the imprecision of the very small studies from which they were recalculated. Several reviews published in 2026 cover adjacent ground, examining multi-omic and molecular predictors of response [121,122], the evidence base for total neoadjuvant therapy and organ preservation [123], and ctDNA during total neoadjuvant therapy [24]. The present article differs from these in its narrower question—whether, and how, molecular evidence should enter the decision to omit surgery—and in proposing a definition and an operational specification that can be written into a protocol, rather than a survey of biomarkers.

Two statements should therefore be made without qualification. Molecular negativity cannot currently be interpreted as proof of cure. And ctDNA should complement, not replace, digital rectal examination, endoscopy, MRI and clinical judgment in deciding whether an operation may be omitted.

## 9. What Evidence Is Needed Before Clinical Implementation

The gap between where the field is and where it needs to be is well defined, which is itself encouraging. It is also already populated: Table 7 lists the studies registered on ClinicalTrials.gov in which ctDNA is intended to inform organ preservation or response assessment after neoadjuvant therapy, and the requirements set out below are written against them.

Ten such studies were identified (Table 7). Seven are active or planned, with target samples of 50 to 470 patients; four are observational cohorts whose primary endpoints are response rates or ctDNA status rather than decision outcomes, and none randomize the watch-and-wait decision itself according to molecular status. The only randomized study, CINTS-R, uses ctDNA to adapt the intensity of neoadjuvant treatment, and its interim report addresses feasibility and safety rather than efficacy [124]. Three studies were withdrawn without enrolling a patient—one for lack of funding, one because the sponsor had already published the data the study was designed to generate, and one after the sponsor decided not to proceed at the participating site—which is a fair measure of how difficult these studies are to mount. Most registered primary completion dates fall in 2027 and 2028.

What is required first is methodological: prospective multicenter cohorts with standardized ctDNA sampling, prespecified timepoints anchored to the treatment schedule rather than to convenience, uniform assay platforms with declared limits of detection, paired leukocyte sequencing, and explicit definitions of molecular response and molecular residual disease. Studies should also report sensitivity, specificity and predictive values at each prespecified timepoint, with the prevalence of residual disease in the population tested, and not hazard ratios alone. Without this, further meta-analyses will continue to produce impressive pooled hazard ratios that cannot be translated into a decision rule [90,91,99].

What is required second is integration. Molecular findings need to be reported alongside standardized anatomical assessment in the same patients and at the same timepoints, so that the four clinical–molecular phenotypes can be populated with real outcome data. International consensus already exists on the outcome measures that organ preservation trials should report—TME-free survival, organ preservation rate, regrowth, salvageability, disease-free and overall survival, and quality of life—and molecular endpoints should be mapped onto that existing framework rather than invented separately [13].

What is required third, and decisively, is interventional evidence in which molecular findings actually influence management. The design we consider most defensible is a de-escalation trial: patients with an anatomical clinical complete response, demonstrable ctDNA shedding at baseline and sustained molecular negativity at restaging would be randomized to standard surveillance or to de-escalated endoscopic and MRI surveillance, with non-salvageable regrowth and distant metastasis as safety endpoints and with surveillance burden, cost and quality of life as the endpoints of benefit. The alternative design—randomizing ctDNA-positive patients with an apparent clinical complete response to immediate TME or to intensified surveillance—is not supported by the present data: with a positive predictive value of 23.5% for recurrence using the most sensitive assay reported so far [94], most patients allocated to proctectomy would already be cured, and since a positive result may signal distant rather than local disease, the logical response to it is complete systemic restaging within an observational cohort rather than a pelvic operation [100]. Feasibility deserves attention in either design. For illustration, if roughly 40% of patients reach a clinical complete response after TNT, as in the OPRA response-grade analysis [19], and between two-thirds and four-fifths of those are molecularly negative with a polymerase chain reaction-based tumor-informed assay [95,97], then three to four patients would have to start TNT for each patient eligible for randomization; with the whole-genome assay, which detected ctDNA at restaging in 17 of 26 patients [94], that number would roughly double. Demonstrating that a biomarker is prognostic is not the same as demonstrating that acting on it improves outcomes; the DYNAMIC trial made exactly this transition in the adjuvant setting, and it is the transition that organ preservation now requires [92]. Cost-effectiveness and quality-of-life endpoints should be embedded from the outset, since the value proposition of precision watch-and-wait is as much about avoided harm and avoided testing as about avoided recurrence [15,48].

## 10. Conclusions

Organ preservation in rectal cancer has advanced far enough that its central weakness is now clearly visible. We select patients for the omission of surgery using criteria that describe the appearance of the tumor bed, and we accept a regrowth rate that tells us those criteria are biologically incomplete. Circulating tumor DNA and the wider family of molecular biomarkers offer a different kind of evidence, and in mismatch repair-deficient disease molecular information already redirects treatment onto a pathway in which surgery is frequently avoided. But the current generation of ctDNA data in this setting comes from a handful of small cohorts, is prognostic rather than actionable, and displays a trade-off between sensitivity and specificity that no assay has yet resolved: with the most sensitive assay, fewer than one in four positive results during surveillance was followed by recurrence, which is not an adequate basis for a decision as irreversible as proctectomy.

The future of rectal preservation will most likely depend not on replacing clinical complete response with molecular response, but on integrating anatomical, clinical and molecular evidence into a more precise definition of treatment response. An integrated complete response, prospectively defined and prospectively tested, is the vocabulary this integration requires; precision watch-and-wait is the strategy it would enable. Its most defensible first application is to test whether surveillance can be safely relaxed in patients whose anatomical and molecular assessments agree, and not to send molecularly positive patients to the operating room. Until the interventional trials are done, the correct posture is neither dismissal nor adoption, but disciplined curiosity: measure the molecular layer systematically, report it transparently, and do not yet let it decide.

## Figures and Tables

**Figure 1:**
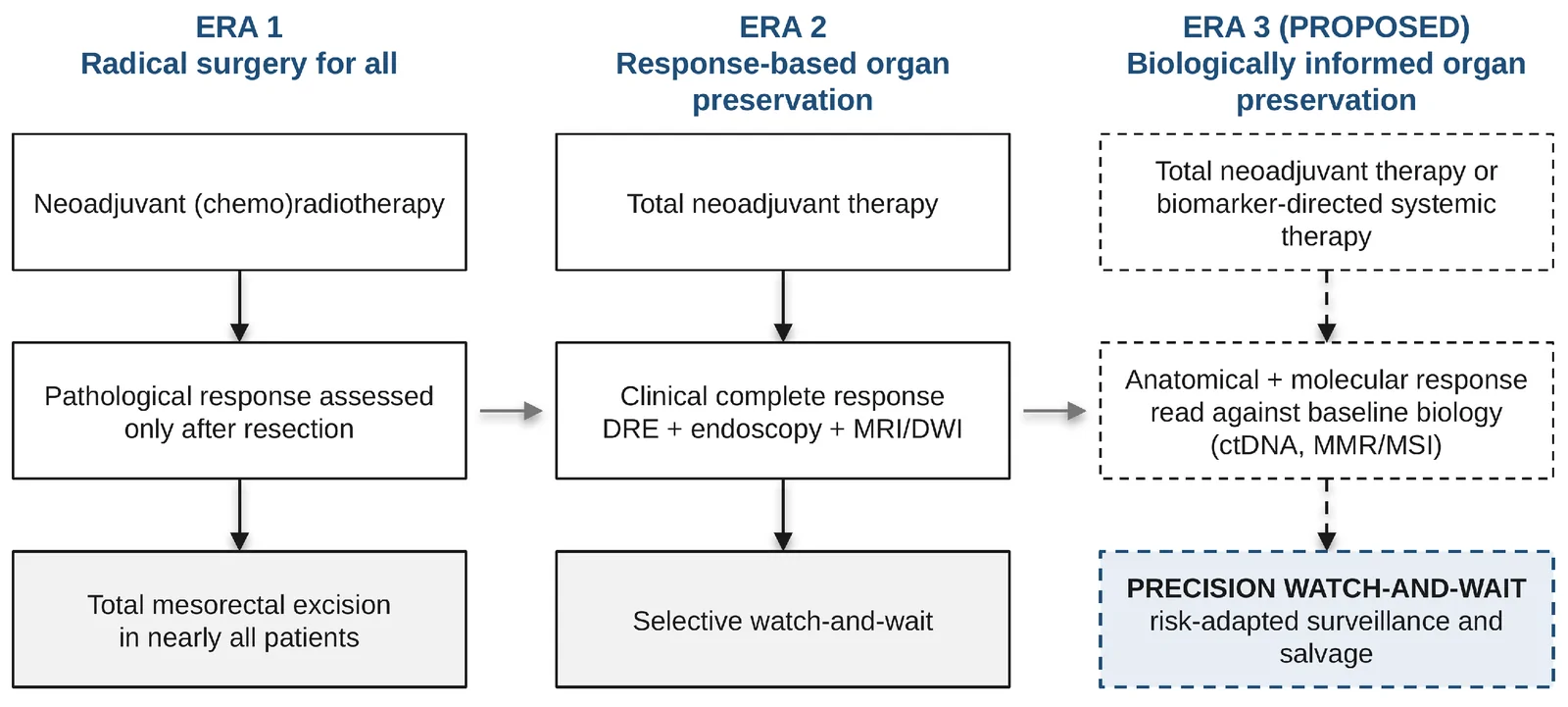
Evolution of rectal cancer management, from radical surgery for all patients to precision watch-and-wait. In the first era, neoadjuvant (chemo)radiotherapy was followed by total mesorectal excision in nearly all patients and response could be assessed only in the resection specimen. In the second era, total neoadjuvant therapy increased complete response rates and made selective watch-and-wait possible. The third era is proposed and investigational, shown with dashed outlines: anatomical and molecular response assessment, read against baseline tumor biology, would support risk-adapted, precision watch-and-wait, but none of its molecular components are yet validated for this decision. Vertical arrows indicate the sequence of steps within each era (dashed in the proposed third era), horizontal arrows the transition from one era to the next, and shaded boxes the resulting management strategy. DRE: digital rectal examination; MRI: magnetic resonance imaging; DWI: diffusion-weighted imaging; ctDNA: circulating tumor DNA; MMR: mismatch repair; MSI: microsatellite instability.

**Figure 2:**
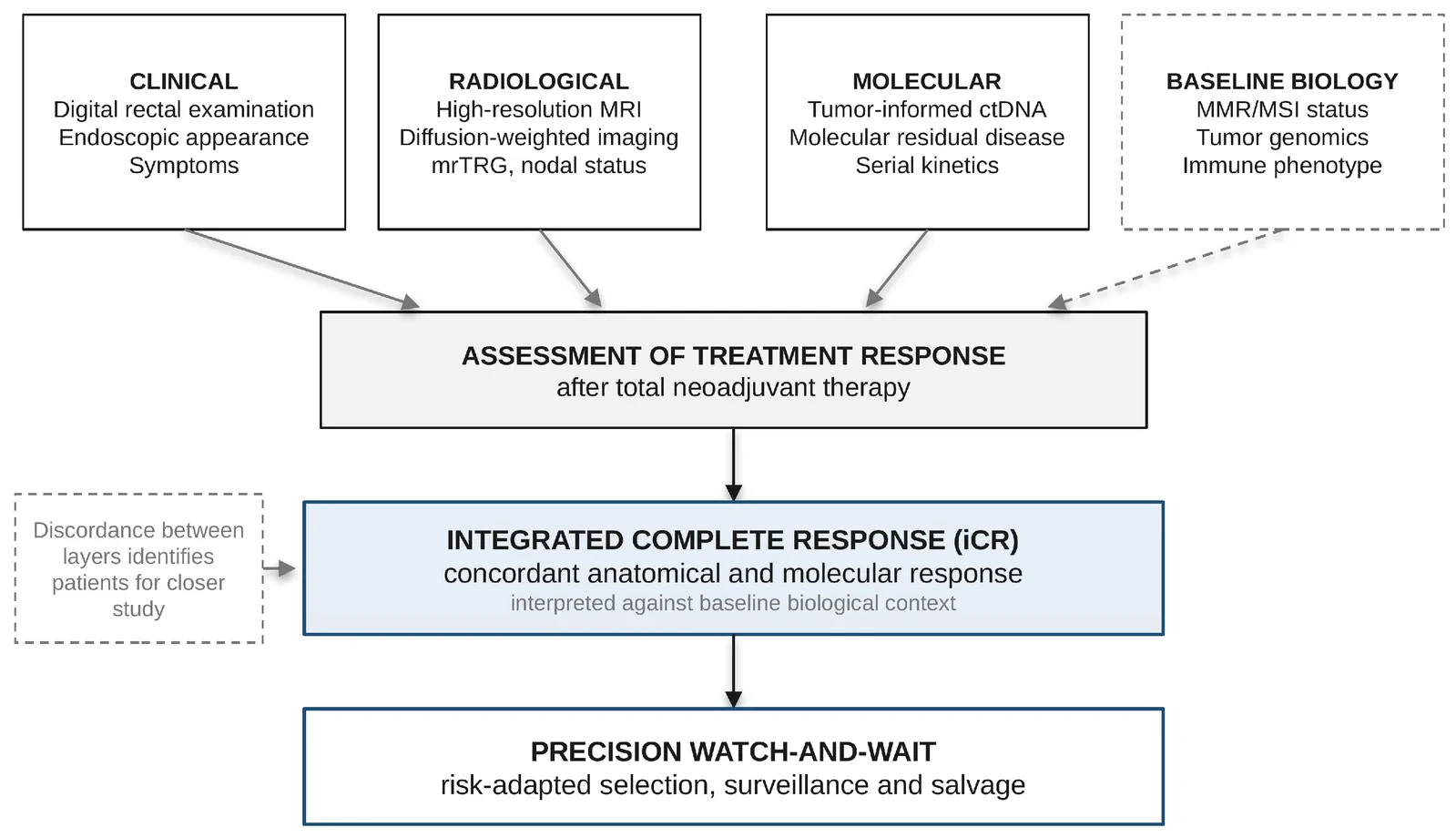
From clinical complete response to an integrated complete response. Two response layers are assessed after total neoadjuvant therapy: anatomical response, comprising clinical (digital rectal examination, endoscopy) and radiological (MRI) assessment, and molecular response. A concordant complete response in both layers is proposed as an integrated complete response, interpreted against baseline biological context—mismatch repair and microsatellite status, tumor genomics, immune phenotype and baseline ctDNA shedding—which modifies the expected response but is not itself a response layer. Discordance between layers identifies the patient groups in which prospective study is most needed. The construct is proposed for research and is not a clinical criterion. MRI: magnetic resonance imaging; mrTRG: magnetic resonance tumor regression grade; ctDNA: circulating tumor DNA; MMR: mismatch repair; MSI: microsatellite instability.

**Table 1:** Principal trials, registries and pooled analyses of organ preservation in rectal cancer.

Study	Design	Population	Main Organ-Preservation Finding	Main Limitation
São Paulo series [9]	Single-center series	265 patients after chemoradiotherapy; 71 (26.8%) with a clinical complete response observed	Five-year overall and disease-free survival of 100% and 92% in the observation group	Pre-MRI era; selection criteria and response definitions not standardized
Maastricht program [36,37]	Prospective single-program cohort	100 patients: 61 with a clinical complete response, 39 near-complete	15 local regrowths, all salvageable; three-year local regrowth-free survival 84.6%	Single program; near-complete responders managed by reassessment or local excision
OnCoRe [43]	Propensity-score matched cohort	129 managed by watch-and-wait; 109 matched pairs	Regrowth in 44 of 129 (34%); three-year colostomy-free survival 74% versus 47%, with no difference in non-regrowth disease-free survival	Observational; matching on few covariates
International Watch & Wait Database [11]	International registry	880 patients with a clinical complete response from 47 institutions	Two-year cumulative incidence of regrowth 25.2%; 88% of regrowths within two years; distant metastasis in 8%	Heterogeneous selection, imaging and surveillance between centers
InterCoRe consortium [42]	Individual participant data meta-analysis	602 patients from 11 studies	Two-year cumulative incidence of regrowth 21.4%, increasing with cT category	Between-study heterogeneity (I^2^ = 61%)
Memorial Sloan Kettering series [20]	Retrospective single-center series	113 managed by watch-and-wait; 136 with a pathological complete response after TME	Rectum preserved in 93 of 113 (82%); all 22 regrowths salvaged; distant metastasis in 36% with versus 1% without regrowth	Retrospective; comparator defined by pathology rather than by clinical response
OPRA [12,18,19]	Randomized phase II (induction versus consolidation chemotherapy) with response-adapted TME or watch-and-wait	324 randomized; 123 complete and 94 near-complete responders offered watch-and-wait	Five-year TME-free survival 39% versus 54%; regrowth in 27 of 123 (22.0%) after a complete and 48 of 94 (51.1%) after a near-complete response	Did not randomize watch-and-wait against TME; disease-free survival compared with historical data
GRECCAR 2 [49,50]	Randomized phase III, local excision versus TME in good responders	145 patients with cT2–T3 low rectal cancer of ≤4 cm and residual lesion ≤2 cm	Superiority of local excision not shown; 26 of 74 required completion TME; five-year oncological outcomes similar	Highly selected tumors; completion surgery added morbidity
NO-CUT [51]	Single-arm phase II	179 patients with mismatch repair-proficient stage II–III disease	47 (26%) entered nonoperative management; 30-month distant relapse-free survival 95% in that group	No randomized comparator
Dostarlimab studies [52,53]	Single-arm phase II	Mismatch repair-deficient locally advanced rectal cancer (rectal cohort, n = 49)	Clinical complete response in all 49 patients who completed treatment; 37 sustained at 12 months	Applies to a small molecular subgroup; follow-up still limited

OnCoRe: Oncological Outcomes after Clinical Complete Response in Patients with Rectal Cancer; OPRA: Organ Preservation in Patients with Rectal Adenocarcinoma; TME: total mesorectal excision; MRI: magnetic resonance imaging.

**Table 2:** Current and emerging tools for response assessment in rectal cancer.

Tool	What It Measures	Current Role	Main Limitation	Evidentiary Maturity
Digital rectal examination	Palpable residual disease in low tumors	Standard	Operator-dependent; rarely documented in standardized form	Established
Endoscopy	Mucosal response and scar morphology	Standard	Cannot exclude submucosal, intramural or nodal residual disease	Established
High-resolution MRI	Anatomical response, mesorectal fascia, nodal status	Standard	Fibrosis and viable tumor share a low T2 signal	Established
Diffusion-weighted MRI	Cellularity-based functional response	Increasing use	Interpretation variability; only moderate reader agreement	Established/promising
Tumor-informed ctDNA	Molecular residual disease and its kinetics	Investigational	Low shedding in localized disease; limited specificity at very low tumor fraction	Promising
Tumor-agnostic ctDNA	Molecular residual disease without tissue input	Investigational	Lower sensitivity; vulnerable to clonal hematopoiesis	Promising
MMR/MSI testing	Intrinsic tumor biology and immunotherapy sensitivity	Standard of care in a defined subgroup	Applies to a small minority of rectal cancers	Established
Tumor genomics	Prognostic and predictive somatic alterations	Selected indications	Little evidence of value for the organ preservation decision	Investigational
Immune signatures	Response-relevant immune microenvironment	Research	No external validation adequate for clinical use	Investigational
Radiomics and integrated AI models	Multimodal quantitative response prediction	Research	Performance degrades on external validation	Investigational

Evidentiary maturity refers to the strength of evidence supporting use in the watch-and-wait decision, not to commercial availability: established, validated and incorporated into guideline-based response assessment; established/promising, part of routine restaging but with performance limited by interpretation variability; promising, prospective data showing a prognostic association but no validation for this decision; investigational, exploratory data only, without adequate external validation. MRI: magnetic resonance imaging; ctDNA: circulating tumor DNA; MMR: mismatch repair; MSI: microsatellite instability; AI: artificial intelligence.

**Table 5:** Clinical–molecular response phenotypes and their potential implications.

Anatomical Response (Clinical and Radiological)	Molecular Response	Possible Interpretation	Current Approach
Clinical complete response	Negative	Deepest apparent response; lowest apparent residual-risk phenotype	Molecular status currently adds nothing to the decision; watch-and-wait is offered on existing anatomical criteria
Clinical complete response	Positive	Possible occult residual local disease, subclinical distant disease, or assay noise	Uncertain; complete local and systemic restaging; prospective evidence required before molecular positivity alters management
Incomplete or near-complete response	Negative	Low-shedding tumor, delayed responder, or fibrosis misread as residual tumor	Standard clinical and radiological assessment remains decisive
Incomplete or near-complete response	Positive	Persistent disease most likely	Standard oncological management, including total mesorectal excision

These categories are conceptual and should not currently be used as standalone treatment algorithms. An uninformative molecular result, such as the absence of detectable ctDNA at baseline, does not place a patient in the molecular-negative column. ctDNA: circulating tumor DNA.

**Table 6:** Proposed operational specification of the integrated complete response for use in research protocols.

Layer	Measurement	Timing Relative to the End of TNT	Positive (Residual Disease) If	Missing or Uninformative Result
Anatomical–clinical [60,62]	Digital rectal examination and flexible endoscopy, recorded with a standardized template and image documentation	8 ± 4 weeks, as in OPRA; repeated after 8–12 weeks in near-complete responders	Any palpable or visible residual mass, ulceration or nodularity (near-complete or incomplete response on three-tier grading [19,39])	Endoscopy not feasible: layer incomplete and an integrated complete response cannot be assigned
Anatomical–radiological [58,59]	High-resolution T2-weighted and diffusion-weighted MRI with structured reporting	Same assessment window as the clinical examination	Residual intermediate T2 signal or restricted diffusion in the tumor bed, or suspicious mesorectal or lateral nodes	Equivocal study: repeat after 8–12 weeks; not scored as complete until resolved
Molecular [79,91,94,95]	Tumor-informed ctDNA assay with a declared limit of detection, declared analytical specificity and fixed plasma volume; paired leukocyte sequencing where the platform requires it	Baseline before TNT to establish shedding; ≥4 weeks after TNT and within 2 weeks of the anatomical assessment; then serially (for example every 3 months for 2 years)	Positive according to the prespecified, analytically validated calling rule of the assay, with the estimated tumor fraction reported alongside the binary call	No detectable ctDNA at baseline (non-shedding tumor) or failed sample: the layer is uninformative rather than negative; an integrated complete response cannot be assigned and the patient is classified by anatomical response alone
Baseline biological context (not a response layer) [52,102]	Mismatch repair and microsatellite status by immunohistochemistry or polymerase chain reaction; baseline ctDNA shedding and tumor fraction; tumor genomics and immune phenotype where available	At diagnosis, before any treatment	Not applicable: context modifies the expected trajectory (for example, mismatch repair deficiency directs treatment to checkpoint blockade) rather than defining response	Mismatch repair or microsatellite status missing: test before treatment; genomics and immune phenotype optional and recorded as not assessed

These specifications are proposals for prospective protocols and are not clinical criteria. TNT: total neoadjuvant therapy; MRI: magnetic resonance imaging; ctDNA: circulating tumor DNA; OPRA: Organ Preservation in Patients with Rectal Adenocarcinoma.

**Table 7:** Studies registered on ClinicalTrials.gov in which circulating tumor DNA is intended to inform organ preservation or response assessment after neoadjuvant therapy for rectal cancer.

Identifier (Acronym)	Sponsor, Country	Design; Planned Sample	Role of Circulating Tumor DNA and Primary Endpoint	Status and Registered Primary Completion (September 2026)
NCT05081024	OHSU Knight Cancer Institute, USA	Observational cohort; 50	Establishing a ctDNA biomarker to improve organ-preserving strategies; primary endpoint: clinical complete response	Recruiting; September 2027
NCT05629442	Massachusetts General Hospital, USA	Observational cohort; 60	ctDNA and organ preservation; primary endpoints: rate of successful nonoperative management and pathological complete response at one year	Not yet recruiting; June 2028
NCT05674422 (GEMCAD-REVEAL)	Grupo Español Multidisciplinar en Cáncer Digestivo, Spain	Observational cohort; 120	ctDNA as a predictor of relapse in patients followed by watch-and-wait or surgery; primary endpoint: ctDNA at two years	Active, not recruiting; April 2025
NCT07209215 (ULTIMATE)	University of California Davis, USA	Non-randomized phase II; 200	ctDNA-informed management versus standard surgery after TNT; primary endpoints: pathological complete response in each arm and disease-free survival	Not yet recruiting; May 2028
NCT05601505 (CINTS-R)	Peking Union Medical College Hospital, China	Randomized phase II; 470	ctDNA-guided intensity of neoadjuvant treatment; primary endpoint: two-year disease-related treatment failure	Recruiting; October 2025
NCT07393048	Beijing Friendship Hospital, China	Observational cohort; 60	Personalized ctDNA molecular residual disease testing to predict the efficacy of neoadjuvant therapy; primary endpoint: complete response rate	Recruiting; December 2028
NCT06328361 (NORPPA-1)	Tampere University Hospital, Finland and Estonia	Single-arm interventional; 200	Nonoperative management after clinical complete response; ctDNA-free survival as a secondary endpoint; primary endpoint: disease-free survival	Recruiting; December 2028
NCT03749083	Massachusetts General Hospital, USA	Observational cohort; 0 enrolled	ctDNA and the likelihood of organ preservation after clinical complete response	Withdrawn (funding)
NCT05356585	Case Comprehensive Cancer Center, USA	Observational cohort; 0 enrolled	ctDNA to monitor the efficacy of total neoadjuvant therapy	Withdrawn (data already published by the sponsor)
NCT07292285 (SNOWW)	University of Colorado, USA	Observational; 0 enrolled	ctDNA to improve selection for watch-and-wait and to detect regrowth during surveillance	Withdrawn (site not opened)

Search of ClinicalTrials.gov performed on 20 September 2026 [23]; the search string and the number of records screened are given in Supplementary File S1 (Table S3). TNT: total neoadjuvant therapy; ctDNA: circulating tumor DNA.

## Data Availability

No new data were created or analyzed in this study. Data sharing is not applicable to this article.

## Supplementary Materials

The following supporting information can be downloaded at: https://www.mdpi.com/article/10.3390/medicina62101865/s1. Supplementary File S1: Search strategies, search dates and record counts; Table S1: PubMed/MEDLINE search; Table S2: Europe PMC search; Table S3: ClinicalTrials.gov search.

## Abbreviations

The following abbreviations are used in this manuscript:

cCR	clinical complete response
CI	confidence interval
ctDNA	circulating tumor DNA
DRE	digital rectal examination
DWI	diffusion-weighted imaging
HR	hazard ratio
iCR	integrated complete response
MMR	mismatch repair
MRI	magnetic resonance imaging
MSI	microsatellite instability
TME	total mesorectal excision
TNT	total neoadjuvant therapy
