# Supplementary material for: Can Molecular Response Redefine Organ Preservation in Rectal Cancer? From Clinical Complete Response to Precision Watch-and-Wait
Source: Medicina (Kaunas). 2026 Sep 26;62(10):1865. doi: 10.3390/medicina62101865 (PMC13707149; doi:10.3390/medicina62101865)
Supplement: Supplementary file 1 [file medicina-62-01865-s001.zip › medicina-4581842-supplementary.pdf]

Supplementary File S1

Can Molecular Response Redefine Organ Preservation in Rectal Cancer? From Clinical Complete Response to Precision Watch-and-Wait

Search strategies, search dates and record counts

All searches were run on 20 September 2026 by one author (A.Y.); the selection of studies was verified by a second author (O.Y.), and disagreements were resolved by discussion. Records were screened by title and abstract, and potentially eligible full texts were assessed. Because this article is a narrative review, no protocol was registered, study selection was not documented in a PRISMA flow diagram and the number of records excluded at each stage was not recorded. Embase, Scopus, Web of Science and the Cochrane Library were not searched; this is stated as a limitation in Section 8 of the manuscript. Searches were supplemented by hand-searching the reference lists of retrieved reviews and by targeted searches for the principal randomized trials and international registries in the field.

Table S1. PubMed/MEDLINE search.

Set	Search string	Records
#1	("Rectal Neoplasms"[Mesh] OR "rectal cancer"[tiab] OR "rectal carcinoma"[tiab] OR "rectal adenocarcinoma"[tiab] OR "rectal tumor"[tiab] OR "rectal tumour"[tiab]) AND ("watch and wait"[tiab] OR "watch-and-wait"[tiab] OR "wait and see"[tiab] OR "wait-and-see"[tiab] OR nonoperative[tiab] OR "non-operative"[tiab] OR "organ preservation"[tiab] OR "organ-preserving"[tiab] OR "organ sparing"[tiab] OR "clinical complete response"[tiab] OR "complete clinical response"[tiab] OR "near-complete response"[tiab] OR "total neoadjuvant therapy"[tiab] OR "total neoadjuvant treatment"[tiab]) AND ("1900/01/01"[dp] : "2026/09/20"[dp])	2597
#2	("Rectal Neoplasms"[Mesh] OR "rectal cancer"[tiab] OR "rectal carcinoma"[tiab] OR "rectal adenocarcinoma"[tiab] OR "rectal tumor"[tiab] OR "rectal tumour"[tiab]) AND ("Circulating Tumor DNA"[Mesh] OR "Cell-Free Nucleic Acids"[Mesh] OR "Liquid Biopsy"[Mesh] OR ctDNA[tiab] OR "circulating tumor DNA"[tiab] OR "circulating tumour DNA"[tiab] OR "cell-free DNA"[tiab] OR cfDNA[tiab] OR "liquid biopsy"[tiab] OR "molecular residual disease"[tiab] OR "minimal residual disease"[tiab]) AND ("1900/01/01"[dp] : "2026/09/20"[dp])	301
#3	("Rectal Neoplasms"[Mesh] OR "rectal cancer"[tiab] OR "rectal carcinoma"[tiab] OR "rectal adenocarcinoma"[tiab] OR "rectal tumor"[tiab] OR "rectal tumour"[tiab]) AND ("DNA Mismatch Repair"[Mesh] OR "Microsatellite Instability"[Mesh] OR "mismatch repair"[tiab] OR dMMR[tiab] OR "microsatellite instability"[tiab] OR MSI-H[tiab] OR "Immune Checkpoint Inhibitors"[Mesh] OR "PD-1"[tiab] OR dostarlimab[tiab] OR nivolumab[tiab] OR pembrolizumab[tiab] OR "checkpoint blockade"[tiab]) AND (neoadjuvant[tiab] OR preoperative[tiab] OR "Neoadjuvant Therapy"[Mesh]) AND ("1900/01/01"[dp] : "2026/09/20"[dp])	410
#4	("Rectal Neoplasms"[Mesh] OR "rectal cancer"[tiab] OR "rectal carcinoma"[tiab] OR "rectal adenocarcinoma"[tiab] OR "rectal tumor"[tiab] OR "rectal tumour"[tiab]) AND (radiomic*[tiab] OR "Artificial Intelligence"[Mesh] OR "artificial intelligence"[tiab] OR "machine learning"[tiab] OR "deep learning"[tiab] OR "Diffusion Magnetic Resonance Imaging"[Mesh] OR "diffusion-weighted"[tiab] OR "tumor regression grade"[tiab] OR "tumour regression grade"[tiab] OR mrTRG[tiab]) AND (response[tiab] OR "complete response"[tiab] OR restaging[tiab] OR "organ preservation"[tiab] OR "watch and wait"[tiab] OR "watch-and-wait"[tiab]) AND (neoadjuvant[tiab] OR preoperative[tiab] OR "Neoadjuvant Therapy"[Mesh]) AND ("1900/01/01"[dp] : "2026/09/20"[dp])	1111
#5	(#1) OR (#2) OR (#3) OR (#4)	3897
#6	(#2) AND ("watch and wait"[tiab] OR "watch-and-wait"[tiab] OR "wait and see"[tiab] OR "wait-and-see"[tiab] OR nonoperative[tiab] OR "non-operative"[tiab] OR "organ preservation"[tiab] OR "organ-preserving"[tiab] OR "organ sparing"[tiab] OR "clinical complete response"[tiab] OR "complete clinical response"[tiab] OR "near-complete response"[tiab] OR "total neoadjuvant therapy"[tiab] OR "total neoadjuvant treatment"[tiab]) (subset used to identify primary studies of circulating tumor DNA in the organ-preservation setting)	85

Table S2. Europe PMC search.

Set	Search string	Records
#1	(TITLE_ABS:"rectal cancer" OR TITLE_ABS:"rectal carcinoma" OR TITLE_ABS:"rectal adenocarcinoma" OR TITLE_ABS:"rectal tumor" OR TITLE_ABS:"rectal tumour") AND (TITLE_ABS:"watch and wait" OR TITLE_ABS:"watch-and-wait" OR TITLE_ABS:"wait and see" OR TITLE_ABS:"wait-and-see" OR TITLE_ABS:nonoperative OR TITLE_ABS:"non-operative" OR TITLE_ABS:"organ preservation" OR TITLE_ABS:"organ-preserving" OR TITLE_ABS:"organ sparing" OR TITLE_ABS:"clinical complete response" OR TITLE_ABS:"complete clinical response" OR TITLE_ABS:"near-complete response" OR TITLE_ABS:"total neoadjuvant therapy" OR TITLE_ABS:"total neoadjuvant treatment") AND (FIRST_PDATE:[1900-01-01 TO 2026-09-20])	2417
#2	(TITLE_ABS:"rectal cancer" OR TITLE_ABS:"rectal carcinoma" OR TITLE_ABS:"rectal adenocarcinoma" OR TITLE_ABS:"rectal tumor" OR TITLE_ABS:"rectal tumour") AND (TITLE_ABS:ctDNA OR TITLE_ABS:"circulating tumor DNA" OR TITLE_ABS:"circulating tumour DNA" OR TITLE_ABS:"cell-free DNA" OR TITLE_ABS:cfDNA OR TITLE_ABS:"liquid biopsy" OR TITLE_ABS:"molecular residual disease" OR TITLE_ABS:"minimal residual disease") AND (FIRST_PDATE:[1900-01-01 TO 2026-09-20])	208
#3	(TITLE_ABS:"rectal cancer" OR TITLE_ABS:"rectal carcinoma" OR TITLE_ABS:"rectal adenocarcinoma" OR TITLE_ABS:"rectal tumor" OR TITLE_ABS:"rectal tumour") AND (TITLE_ABS:"mismatch repair" OR TITLE_ABS:dMMR OR TITLE_ABS:"microsatellite instability" OR TITLE_ABS:"MSI-H" OR TITLE_ABS:"PD-1" OR TITLE_ABS:dostarlimab OR TITLE_ABS:nivolumab OR TITLE_ABS:pembrolizumab OR TITLE_ABS:"checkpoint blockade" OR TITLE_ABS:"checkpoint inhibitor") AND (TITLE_ABS:neoadjuvant OR TITLE_ABS:preoperative) AND (FIRST_PDATE:[1900-01-01 TO 2026-09-20])	382
#4	(TITLE_ABS:"rectal cancer" OR TITLE_ABS:"rectal carcinoma" OR TITLE_ABS:"rectal adenocarcinoma" OR TITLE_ABS:"rectal tumor" OR TITLE_ABS:"rectal tumour") AND (TITLE_ABS:radiomic* OR TITLE_ABS:"artificial intelligence" OR TITLE_ABS:"machine learning" OR TITLE_ABS:"deep learning" OR TITLE_ABS:"diffusion-weighted" OR TITLE_ABS:"tumor regression grade" OR TITLE_ABS:"tumour regression grade" OR TITLE_ABS:mrTRG) AND (TITLE_ABS:response OR TITLE_ABS:restaging OR TITLE_ABS:"organ preservation" OR TITLE_ABS:"watch and wait" OR TITLE_ABS:"watch-and-wait") AND (TITLE_ABS:neoadjuvant OR TITLE_ABS:preoperative) AND (FIRST_PDATE:[1900-01-01 TO 2026-09-20])	1033
#5	(#1) OR (#2) OR (#3) OR (#4)	3556
#6	(#2) AND (TITLE_ABS:"watch and wait" OR TITLE_ABS:"watch-and-wait" OR TITLE_ABS:"wait and see" OR TITLE_ABS:"wait-and-see" OR TITLE_ABS:nonoperative OR TITLE_ABS:"non-operative" OR TITLE_ABS:"organ preservation" OR TITLE_ABS:"organ-preserving" OR TITLE_ABS:"organ sparing" OR TITLE_ABS:"clinical complete response" OR TITLE_ABS:"complete clinical response" OR TITLE_ABS:"near-complete response" OR TITLE_ABS:"total neoadjuvant therapy" OR TITLE_ABS:"total neoadjuvant treatment")	78

Table S3. ClinicalTrials.gov search.

Field	Query	Records
Condition	rectal cancer OR rectal neoplasms OR rectal adenocarcinoma	
Other terms	(ctDNA OR "circulating tumor DNA" OR "circulating tumour DNA" OR "cell-free DNA" OR "liquid biopsy" OR "molecular residual disease") AND ("organ preservation" OR "watch and wait" OR "watch-and-wait" OR "wait and see" OR "nonoperative" OR "non-operative" OR "clinical complete response")	
Retrieved	All records were screened by title, design and outcome measures; studies in which circulating tumor DNA is intended to inform organ preservation or response assessment after neoadjuvant therapy were retained and are listed in Table 7 of the manuscript.	36 retrieved; 10 retained

Note. Europe PMC indexes MEDLINE records as well as preprints and additional full-text sources, so the two databases overlap substantially; counts are reported for each database separately and were not deduplicated.
